# Intrauterine Growth Retardation Fetus with Trisomy 16 Mosaicism

**DOI:** 10.1155/2014/739513

**Published:** 2014-05-14

**Authors:** Takol Chareonsirisuthigul, Suchin Worawichawong, Rachanee Parinayok, Patama Promsonthi, Budsaba Rerkamnuaychoke

**Affiliations:** ^1^Department of Pathology, Faculty of Medicine Ramathibodi Hospital, Mahidol University, Bangkok 10400, Thailand; ^2^Department of Obstetrics and Gynecology, Faculty of Medicine Ramathibodi Hospital, Mahidol University, Bangkok 10400, Thailand

## Abstract

Fetal trisomy 16 is considered uniformly lethal early in gestation. It has been reported to be associated with the variability of clinical features and outcomes. Mosaic trisomy 16 leads to a high risk of abnormality in prenatal cases. Intrauterine growth retardation (IUGR) is a common outcome of mosaic trisomy 16. Herein, we report on the case of Thai male IUGR fetus with trisomy 16 mosaicism. The fetal body was too small. Postmortem investigation of placenta revealed the abnormality including small placenta with furcated cord insertion and single umbilical cord artery. Cytogenetic study demonstrated trisomy 16 that was found 100% in placenta and only 16% in the fetal heart while other organs had normal karyotype. In addition, cardiac and other internal organs examination revealed normal morphology.

## 1. Introduction

Trisomy 16 is the most commonly observed trisomy in spontaneous abortuses, accounting for over 30% of the autosomal trisomies [1–4]. Trisomy 16 mosaicism diagnosed postnatally has rarely been reported at amniocentesis and is even more exceptionally seen in live borns [3, 5–7]. Full trisomy 16 has never been unequivocally diagnosed at birth. The major cause of trisomy 16 is an error during mechanism of mitotic nondisjunction or anaphase lag and reduction to disomy. As with many trisomic conceptuses, some full trisomy 16 embryos can undergo rescue, with the risk of residual mosaicism and uniparental disomy (UPD) for chromosome 16 in the surviving fetus. UPD can have an adverse effect on fetal development through imprinted genes, and placental mosaicism can be a cause of placental insufficiency, which can disturb fetal development [8, 9].

Mosaic trisomy 16 is associated with a high risk of abnormal outcome, cases commonly exhibiting intrauterine growth retardation (IUGR), fetal-death-in-utero, preeclampsia, preterm delivery, neonatal death, developmental delay, congenital heart defect, and other minor anomalies [5]. Nevertheless more recent reports may suggest that a significant proportion of prenatally detected patients with mosaic trisomy 16 have mild phenotype and good outcome [10, 11]. This case study gives a detailed account of the detection of mosaic trisomy 16 by which several tissues from various organs were investigated and sent for cytogenetic analysis.

## 2. Case Presentation

A 36-year-old Thai woman had the underlying disease of hypertension. The previous gestation lasted for 4 years and her first child was the female who was born by cesarean section due to preeclampsia. She received the first antenatal care at 5 weeks of gestational age. The investigation results showed that the mother had heterozygous beta-thalassemia (the father had heterozygous HbE). Sonographic examination at 18 weeks demonstrated multiple abnormal findings including intrauterine growth retardation, single umbilical cord artery, and hypospadias. Due to the abnormal ultrasonographic study, cytogenetic analysis was performed using amniotic fluid. The fetal karyotype was mos 47,XY, +16/46,XY (30%/70%). Sonographic examination at 29 weeks confirmed intrauterine growth restriction (femoral length was 39 mm, in accordance with a GA of 22 weeks). At this point, considering the combined findings from cytogenetic and ultrasound studies, the prediction for fetal prognosis was poor and the prospective parents were informed of this. The parents selected to terminate pregnancy. The procedure was done at 30 weeks and followed by postmortem and cytogenetic studies.

**Figure 1:**
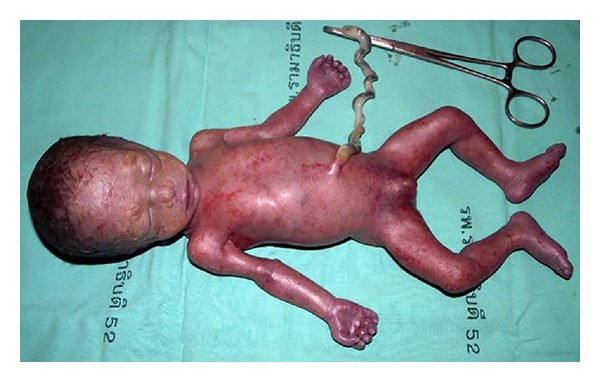
Gross anatomical description: the body of Thai male was symmetrical small for gestational age. The external appearance showed no dysmorphic feature. Contracture of hands and rocket bottom feet, genital hypoplasia, and small placenta with furcated cord insertion and single umbilical artery were noticeable.

The body was the 30 weeks Thai male fetus. His body was symmetrical small for gestational age and resembles for the weight of fetus at GA 25-26 weeks. The external appearance showed no dysmorphic feature. However, contracture of hands and rocket bottom feet were detected. Genital hypoplasia and small placenta with furcated cord insertion and single umbilical artery were noticeable (Figure 1). Other internal organs showed no malformation. The microscopic examination of placenta showed subchorionic perivillous fibrin deposit approximately 10–15% of the placental area. The remaining parenchyma showed normal villous architecture.

Specimens for karyotyping were obtained from the fetal and the placental tissues. The karyotypes demonstrated 47,XY,+16 for 100% in 50 metaphases of the placenta, but only 16% (10/119) in the heart. However, other specimens from cord blood, heart blood, the lung, the liver, the intestine, the brain, and the kidney were normal karyotype, 46,XY (Table 1).

## 3. Discussion

The number of reports of trisomy 16 mosaicism in late pregnancy or at birth is presently too small to define a characteristic phenotype. However, IUGR, aortic coarctation, congenital heart defect, intestinal atresia, craniofacial dysmorphisms, and various anomalies have been reported [5–7, 12–16].

According to our case, the postmortem investigation of placenta revealed the abnormalities including small placenta with furcated cord insertion and single umbilical cord artery as previously detected in the prenatal ultrasonography. The abnormalities of the placenta might be the subsequences of trisomy 16 that confined within this organ. Therefore, combined with the history of maternal preeclampsia, uteroplacental insufficiency was considered as the major cause of IUGR in this case.

The fetal body was examined as well. The body weight was too small for gestational age and resembles the fetus in 25-26 weeks of age. Mosaic trisomy 16 might cause the little effect to the fetus because they were identified in small proportion and confined to the heart only. In addition, the postmortem cardiac examination revealed normal cardiac morphology. Therefore, this symmetric IUGR could be described by the uteroplacental insufficiency as previously discussed.

In conclusion, our study revealed that this 30-week male fetus had trisomy 16, mosaic type. The trisomy 16 confined within the placenta in 100% and only 16% in the heart of fetus. The other organs revealed normal karyotype. The phenotypic features were small placenta with furcated cord insertion and single umbilical cord artery. The fetal body showed symmetrical small gestational age without internal organ malformation.

## Figures and Tables

**Table 1:** Cytogenetic analysis: several tissues from various organs were sampled and sent for karyotyping studies.

Specimen	Culture time (days)	Cytogenetic result	Total cell (cells)
Cord blood	3	46,XY	103
Heart blood	3	46,XY	84
Lung	11	46,XY	107
Liver	11	46,XY	117
Intestine	11	46,XY	106
Heart	12	mos 47,XY, +16 [19]/46,XY [100]	119
Brain	12	46,XY	100
Kidney	12	46,XY	120
Placenta	17	47,XY,+16	50
Cord	—	Culture failure	—
